# Investigation of Accuracy and Influence Factors of Predicting Lenticule Thickness in Small Incision Lenticule Extraction by Machine Learning Models

**DOI:** 10.3390/jpm13020256

**Published:** 2023-01-30

**Authors:** Huihang Wang, Shaobin Zheng, Shumin Tang, Xiaojuan Zhang, Yingying Chen, Yihua Zhu

**Affiliations:** Department of Ophthalmology, The First Affiliated Hospital of Fujian Medical University, No. 20 Chazhong Road, Fuzhou 350005, China

**Keywords:** SMILE, lenticule thickness, machine learning, Random Forest

## Abstract

Small-incision lenticule extraction (SMILE) is a safe and effective surgical procedure for refractive correction. However, the nomogram from the VisuMax femtosecond laser system often overestimates the achieved lenticule thickness (LT), leading to inaccurate estimation of residual central corneal thickness in some patients. In order to improve the accuracy of predicting achieved LT, we used machine learning models to make predictions of LT and analyze the influencing factors of LT estimation in this study. We collected nine variables of 302 eyes and their LT results as input variables. The input variables included age, sex, mean K reading of anterior corneal surface, lenticule diameter, preoperative CCT, axial length, the eccentricity of the anterior corneal surface (E), diopter of spherical, and diopter of the cylinder. Multiple linear regression and several machine learning algorithms were employed in developing the models for predicting LT. According to the evaluation results, the Random Forest (RF) model achieved the highest performance in predicting the LT with an R^2^ of 0.95 and found the importance of CCT and E in predicting LT. To validate the effectiveness of the RF model, we selected additional 50 eyes for testing. Results showed that the nomogram overestimated LT by 19.59% on average, while the RF model underestimated LT by −0.15%. In conclusion, this study can provide efficient technical support for the accurate estimation of LT in SMILE.

## 1. Introduction

Small incision lenticule extraction (SMILE) was first reported by Sekundo et al. and Shah et al. in 2011 as a safe and effective treatment of myopia and myopic astigmatism [1,2]. Compared with other refractive surgery, SMILE is an Integrated surgery that involves the creation of an intra-stromal lenticule and a peripheral incision in one step using a femtosecond laser and manual extraction of the lenticule ultimately. A femtosecond laser can rapidly scan corneal stroma with a pulse frequency of hundreds of kilohertz and a small distance between adjacent pulses. In this way, SMILE surgery can avoid or minimize errors associated with excimer laser ablation, such as stromal hydration [3], laser fluence [4,5,6], relative humidity, environmental temperature, and so on [7]. In general, the accuracy and safety of SMILE surgery are not inferior to other excimer laser surgeries [8,9]. However, previous studies have reported that there was still a difference between the predicted LT and the achieved LT. Luft et al. [10] found that the achieved LT was always thinner than the predicted LT, especially with higher myopic correction. Reinstein et al. [11] also detected a systematic overvalue of central LT of approximately 8 μm.

Nevertheless, the prediction of LT is mainly by referencing the nomogram provided by the VisuMax femtosecond laser system. Several studies suggested nomograms for SMILE to be conducted [12,13,14]. However, they were focused only on the influence of spherical, cylindrical, or lenticule diameter (LD) on the change of LT but ignored the influence of other potential variables. Furthermore, the linear regression analysis was generally used to predict LT in previous studies but with other arithmetics. In addition, the nomogram development for SMILE has not been broadly studied yet. 

With the development of artificial intelligence (AI), it has become more and more popular in the medical field [15]. In ophthalmology, AI has been applied extensively to diagnose ophthalmological diseases, such as cataracts, glaucoma, age-related macular degeneration, and diabetic retinopathy [16]. Recently, Tong et al. [17] applied the multi-layer perceptron (MLP) algorithm to train nomogram models for SMILE. However, there was no comparison with the other algorithms in their study, and this study focused more on reducing the postoperative refractive error than the lenticule thickness error. Fang et al. [18] found that the lenticule thickness predicted by the nomogram exceeds the achieved lenticule thickness by approximately 10%, but it’s just a correction.

It is well known that the residual central corneal thickness (CCT) after SMILE is a significant indication of whether surgery can be performed, and the most crucial point is the accurate prediction of the LT. In order to make the prediction more accurate, we included a large number of patients, and multiple prediction models were trained based on these data. The aim of this study was to explore the variables that affect the LT and use these variables to train AI prediction models, find out the best one by comparison, and then use the validation data to verify it.

## 2. Materials and Methods

**Figure 1:**
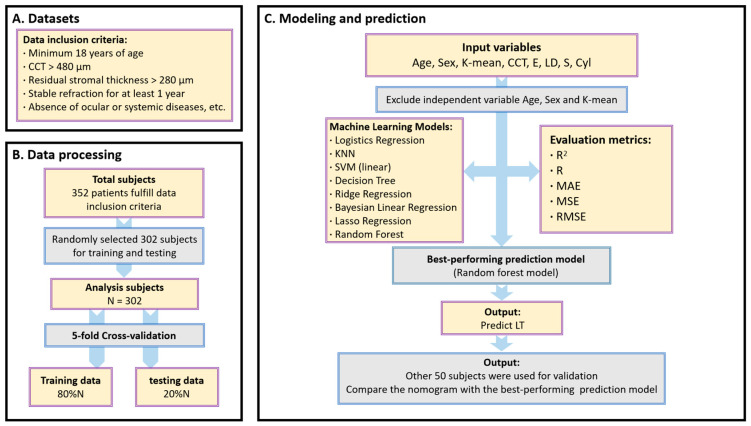
Flowchart of our proposed method. (**A**). Datasets. (**B**). Data processing. (**C**). Machine learning models used to predict the lenticule thickness and compare with the nomogram. The best-performing prediction model was applied to predict the lenticule thickness and use 50 subjects for validation. K-mean = mean K reading; CCT = central corneal thickness; E = eccentricity of the anterior corneal surface; LD = lenticule diameter; S = Sphere; Cyl = Cylinder; LT: lenticule thickness; SVM: support vector machine; KNN: K-Nearest Neighbor; R: the coefficient of determination; MAE: mean absolute errors; MSE: mean squared errors; RMSE: root mean square error; N: number of patients.

This study included 352 consecutive patients who underwent a SMILE procedure at the Eye Center, the First Affiliated Hospital of Fujian Medical University, from March 2022 to September 2022. This study followed the tenets of the Declaration of Helsinki and was approved by the Ethics Committee of the First Affiliated Hospital of Fujian Medical University. Written informed consent was obtained from the subjects before participating in this study. Inclusion criteria included the following: minimum 18 years of age, minimum central corneal thickness (CCT) of 480 mm, calculated residual stromal thickness greater than 280 mm, stable refraction for at least 1 year, absence of ocular or systemic diseases, and reproductive status of not being pregnant or breastfeeding. Patients who wore soft contact lenses were instructed to stop wearing them for at least 1 week before measurement (Figure 1).

Each patient underwent an ophthalmologic examination, including diopter of the sphere (S), diopter of the Cylinder (Cyl), intraocular pressure (IOP), slit-lamp examination, scanning laser ophthalmoscope (SLO, Heidelberg Engineering, Heidelberg, Germany), optical biometry (Lenstar LS900, Haag Steit AG, Koeniz, Switzerland) and Pentacam imaging (Oculus Optikgeräte GmbH, Wetzlar, Germany).

### 2.1. Surgical Procedure

All SMILE procedures were performed by the same surgeon (ZSB) using the VisuMax femtosecond laser system (Carl Zeiss Meditec AG, Jena, Germany) with a 500-kHz repetition rate. All patients went under topical anesthesia (Alcaine; AlconCouvreur n.v., Puurs, Belgium) instilled 2 or 3 times. The laser cut energy index was 155 nJ; the intended cap thickness was 120 μm; the programmed optical zone diameter was between 6.0 and 6.8 mm, and the diameter of the cap was 1 mm larger than the diameter of the lenticule. The optical zone was selected based on the pupil diameter and percent tissue alert (PTA). After the creation of the lenticule, an incision of approximately 2 mm in length was created at the 11 o’clock position for lenticule extraction. After surgery, one drop of dexamethasone steroid (Tobradex; Alcon Laboratories, Fort Worth, TX) was placed in each eye. A recommended nomogram from VisuMax femtosecond laser system was implemented for all subjects to predict LT.

### 2.2. Postoperative Treatment

Patients were instructed to wear plastic shields for 7 nights. The standard postoperative treatment was levofloxacin eye drops (Cravit; Santen Pharmaceutical Co., Ltd., Osaka, Japan) 4 times a day after surgery for 7 days, fluorometholone eye drops (Santen Pharmaceutical Co., Ltd., Osaka, Japan) at 0.1% 4 times a day for 2 weeks, and preservative-free artificial tears 4 times a day for a month. The patients were followed up at 1 day, 1 week and 1 month, and 3 months; the optometry, visual acuity, and IOP were examined at each visit. Pentacam scanning was performed at the 1-day, 1-month, and 3-month postoperative visits.

### 2.3. Achieved LT Calculation

The achieved LT data were calculated by comparing the pre- and postoperative examinations with Pentacam software. The rotating Pentacam Scheimpflug camera measures corneal thickness normal to the anterior surface tangent [19]. The pachymetry values were provided at 3 points [20], including the corneal vertex, pupil center, and the thinnest point. During the examination, the automatic release mode was used [21]. In this study, the intended treatment center was the corneal vertex. Since the position of the thinnest point of the cornea varies greatly from person to person, the corneal vertex and pupil center were selected as the two locations to calculate the achieved LT.

### 2.4. Surgical Refractive Correction

According to the preoperative examination of the computer optometry, mydriatic optometry, and comprehensive optometry design, the expected correction refraction, included S, Cyl, and LD, and they were input into the VisuMax femtosecond laser system; the surgical correction was performed according to these data.

### 2.5. Statistical Analysis

According to the preoperative examination of the computer optometry, mydriatic optometry, and comprehensive optometry design, the expected correction refraction, included S, Cyl, and LD, and they were input into the VisuMax femtosecond laser system; the surgical correction was performed according to these data.

In our study, only one eye for each patient was randomly selected and included for statistical analysis to ensure that the measurements from the eyes could be treated independently [22]. In this study, to build the prediction model, the input variables were age (in years), sex (“1” represents male, while “2” represents female), mean K reading of anterior corneal surface (K-mean, in diopters), LD (in mm), preoperative CCT (in μm), axial length (AL, in mm), the eccentricity of the anterior corneal surface (E), S (in diopter) and Cyl (in diopter), and the target output was the predicted LT (in μm). The *E* is calculated using the following formula, where the *Q* is the *Q* value on the anterior surface of the cornea:

(1) $$E=\sqrt{-Q}$$

Eight types of supervised machine learning models were implemented in our study based on Logistics Regression Model, K-Nearest Neighbor (KNN) model, Support Vector Machine (SVM) regression models, Decision Tree Regression, Ridge Regression (l2 regularization), Bayesian Linear Regression, Lasso Regression (l1 regularization), and Random Forest model. Then, a 5-fold cross-validation [23] scheme randomly divided all data into 5 groups, including 4 groups (80%) used as training data and one group (20%) used as validation data. This process was repeated 5 times so that all data were validated by this model, which allowed better prediction of the overall sample and prevented overfitting. During the fitting process, AL was finally excluded from our final model because it could lead to the problem of collinearity and render the final model unsolvable. Furthermore, including AL did not yield better results. Thus, the final model used to estimate *LT* was as follows:

(2) $$LT_{predicted}=f\left(Age,Sex,Kmean,E,CCT,LD,S,Cyl\right),$$

To verify the efficacy of the machine learning models, a multiple linear regression model was also created [24,25]. Pearson correlation analyses among all the variables. The performance of the machine-learning prediction algorithms developed from the training data was assessed using the testing data by calculating the R^2^ value, R value, mean absolute error (MAE), mean squared error (MSE), and root mean square error (RMSE), the best performance model compared with the nomogram from VisuMax femtosecond laser system using validation data by paired *t*-tests. All statistical analyses were performed using the PyCharm (Edition 2020.1.2 x64) embedded by the Python (Python Software Foundation) software (Version 3.8) under the Windows 10 system, and the level of statistical significance was set at *p* < 0.05.

## 3. Results

**Figure 2:**
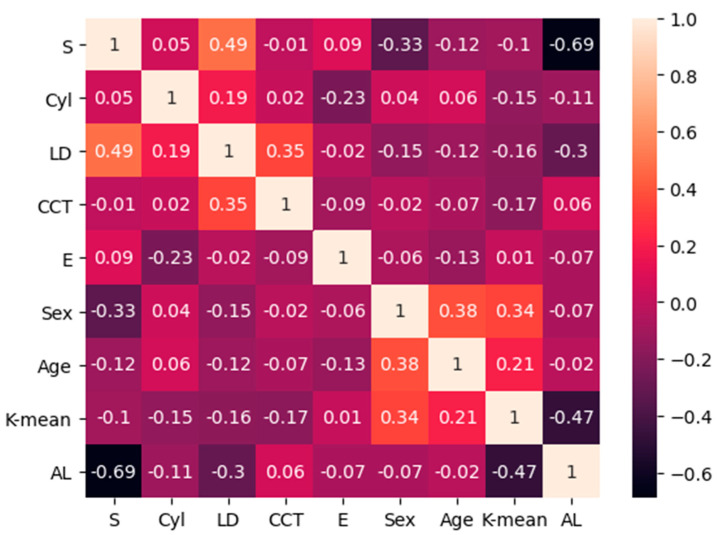
This figure shows the correlation between all variables. The absolute value 0.8–10 is an extreme high correlation, 0.6–0.8 is a high correlation, 0.4–0.6 is a medium correlation, 0.2–0.4 is a weak correlation, and 0–0.2 is no correlation.

In total, 189 males (53.69%) and 163 females (46.31%) were included in this study. The average age of all subjects was 22.87 ± 5.92 years. The mean S was −5.75 ± 2.15 D and ranged from −1.5 D to −10.00 D. The mean Cyl was −0.96 ± 0.74 D and ranged from 0 to −3.75 D. The detailed information is provided in Table 1. Via Pearson correlation analyses among all variables included in the study, we found that there was a high linear correlation between the AL and the S (r = 0.69), a medium linear correlation between AL and K-mean (r = −0.47), and a medium linear correlation between S and LD (r = −0.49); the others showed weak or no correlation; the detailed information is provided in Figure 2. The AL was finally excluded because it could lead to the problem of collinearity. We randomly selected 302 subjects for training and testing data of the machine learning model, and the other 50 subjects were used for validation data. Significant correlations were found between several input variables and *LT*, but age, sex, and K-mean were not. The R^2^ value of the multiple linear regression model was 0.87, as determined by the following equation:

(3) $$LT_{predicted}=-187.07-11.26\times S-12.17\times Cyl\;+29.40\times LD+0.046\times CCT-12.19\times E(P<0.05),$$

Table 2 shows the performance results of eight machine learning models and the multiple linear regression model using 302 subjects. The results show that three machine learning models had a worse predictive ability than the multiple linear regression model; according to R^2^, the half of machine learning models had a similar predictive ability to the multiple linear regression model and only the Random Forest model achieved significantly better performance. According to Table 2, Random Forest performs optimally in four indicators.

**Figure 3:**
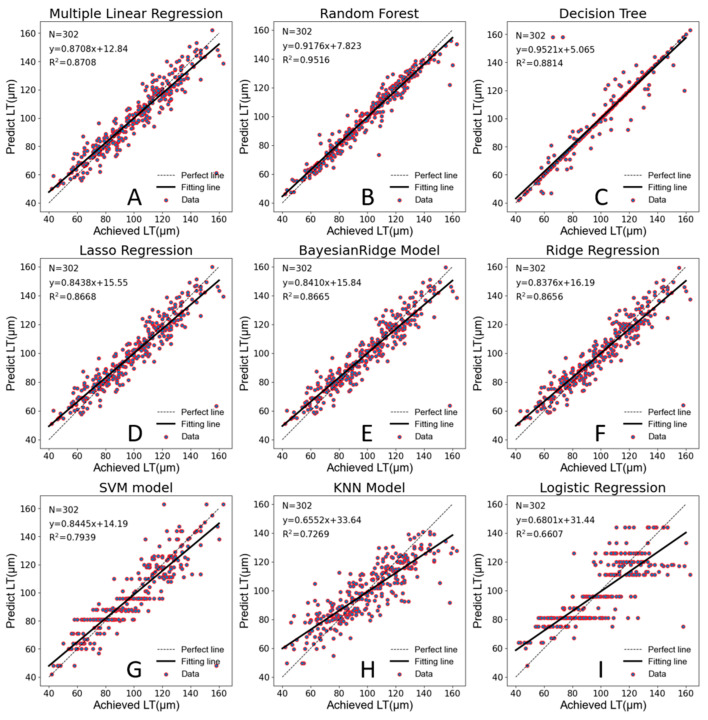
Scatterplot of predicted LT vs. achieved LT. The solid line represents the fitting line. The dashed line represents the perfect line without error prediction. (**A**). Multiple linear regression. (**B**). Random forest. (**C**). Decision tree. (**D**). Lasso regression. (**E**). BayesianRidge model. (**F**). Ridge regression. (**G**). SVM model. (**H**). KNN model. (**I**). Logistic regression.

In this study, in order to visualize the prediction effect and prediction accuracy of each model, we conducted robust linear regression analysis with the achieved LT as the abscissa and the predicted LT as the ordinate. As shown in Figure 3, the Random Forest model has the highest prediction accuracy (R^2^ = 0.9516), and most scatter plots fall along the perfect correlation regression line. The Decision Tree Regression appeared to have great prediction accuracy on the surface, but it actually had serious overfitting. The five-fold cross-validation also confirmed that this model lacked a serious generalization ability.

### 3.1. Model Variables

A total of eight variables were included in the multiple linear regression model. As is known to all [12,13,14], S, Cyl, and LD are the main factors affecting LT, which were the most important variables for predicting LT according to the nomogram from VisuMax femtosecond laser system. In this study, we accidentally found that CCT and E were also significantly correlated with LT, while the other three variables (age, sex, and K-mean) showed no significant correlation, which was eliminated in the prediction model. As shown in Table 3, CCT was positively correlated with LT, while E was negatively correlated with LT.

**Figure 4:**
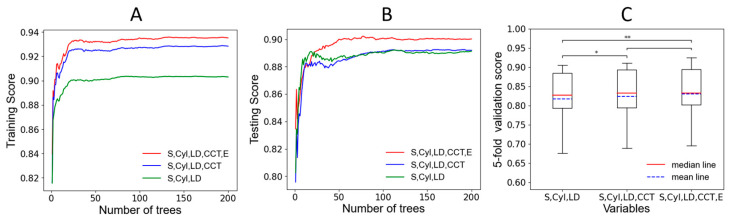
S: Sphere; Cyl: Cylinder; LD: lenticule diameter; CCT: central corneal thickness; E: eccentricity of the anterior corneal surface; * indicates that the *p*-value less than 0.05; ** indicates that the *p*-value less than 0.01. (**A**). The training score changes with the number of trees for three groups of variables. (**B**). The testing score changes with the number of trees for three groups of variables. (**C**). The five-fold cross-validation and the *t*-tests.

To further explore the importance of CCT and E in machine learning models, we selected the best-performing Random Forest model for validation. We included three variables (S, Cyl, LD), four variables (S, Cyl, LD, CCT), and five variables (S, Cyl, LD, CCT, E) into the model, respectively, and observed the performance of the model in the training data and testing data. As shown in Figure 4A,B, the abscissa is the number of trees in the Random Forest model (the more trees, the more stable the model), and the ordinate is the score of the training or testing data; we can find that including CCT and E to the Random Forest model will improve the accuracy of the model in training and testing data. Then we used five-fold cross-validation to verify the accuracy of the three stable Random Forest models and tested them by paired *t*-tests (Figure 4C). There were significant differences between three variables (S, Cyl, LD) and four variables (S, Cyl, LD, CCT) (*p* < 0.05), and there were extremely significant differences between three variables (S, Cyl, LD) and five variables (S, Cyl, LD, CCT, E) (*p* < 0.01).

### 3.2. Prediction Results

**Figure 5:**
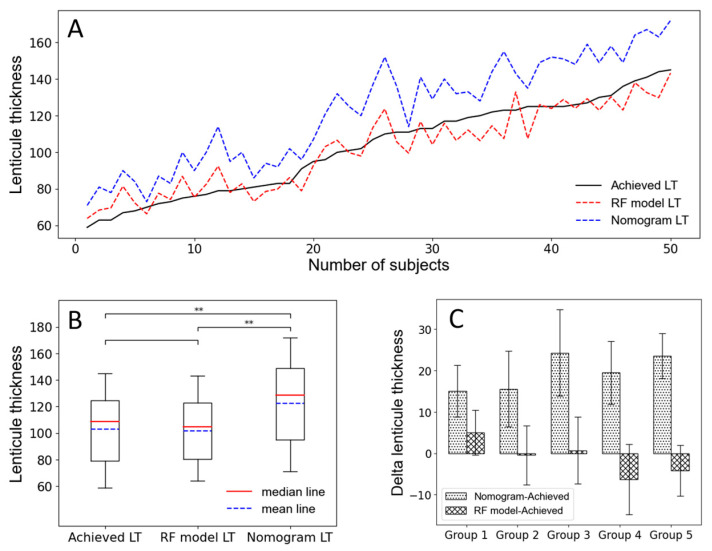
RF: Random Forest; LT: lenticule thickness; ** indicates that the *p*-value is less than 0.01. (**A**). The LT changes with the number of subjects. (**B**). The paired *t*-test on the achieved LT and the predicted LT by the Random Forest model. (**C**). The Delta LT for the nomogram and RF, respectively, for five groups.

In this study, we collected 50 subjects as the verification data. The 50 subjects were independent of the training and testing data, which could more truly evaluate the accuracy of the model’s prediction. As Figure 5A shows, we arranged the 50 subjects according to the achieved LT value from low to high as abscissa can find that the predicted LT by the Random Forest model is very close to the achieved LT, while the nomogram from VisuMax femtosecond laser system is often overestimated, which is similar to previous studies [10,19,26]. Then, we took a paired *t*-test on the achieved LT and the predicted LT by the Random Forest model, as Figure 5B shows, and found that there was no significant difference between them (*p* > 0.05), indicating that the Random Forest model had excellent predictive accuracy. Correspondingly, there is an extremely significant difference between achieved LT and predicted LT by the nomogram (*p* < 0.01). The 50 subjects were divided into five groups according to the achieved LT from low to high, with 10 subjects in each group, to observe the estimated deviations (Delta) of achieved LT by nomogram and Random Forest model. As Figure 5C shows, we found that for the nomogram, the higher value of the achieved AL, the greater overestimation, while the Random Forest model is on the contrary. We converted the overestimate or underestimate values to percentages (the details are shown in Table 4) and found that the nomogram was overestimated by 19.59%, on average.

## 4. Discussion

Myopia is the most common cause of vision loss, with an uncorrected refractive error being the leading ocular disorder, causing visual impairment worldwide [27]. Now a global public health burden [28,29], myopia has become significantly more prevalent across East Asia [30,31]. In order to realize higher visual quality and get rid of the shackles of glasses, more and more people choose refractive surgery to correct refractive errors [32]. SMILE, as a relatively new procedure, is gaining more popularity, especially after its FDA approval for myopia in 2016 and for astigmatism in 2018. In SMILE surgery, the refractive error is corrected by the intrastromal lenticule extraction, so the accuracy of the estimate LT is one of the key points of the SMILE procedure. In the current clinical work, we always reference the nomogram from VisuMax femtosecond laser system to predict the postoperative residual CCT in most cases and take this as the standard to judge whether the refractive error can be fully corrected. However, after a period of clinical practice, we found that the nomogram always overestimated the LT, resulting in an underestimation of the residual CCT. According to this standard, for some patients who could have been fully corrected for refractive error, in order to retain enough residual CCT, we often choose to undercorrect, which has a certain impact on the uncorrected visual acuity and satisfaction of patients. In previous studies, some researchers have also found that the nomogram was not accurate. Liang et al. [13] suggested adding an 11% correction of SE to the nomogram for SMILE surgery; Zhou et al. [26] adjusted the mean treated SE up to −6.30 ± 2.00 D when the mean preoperative SE was −5.96 ± 1.97 D in SMILE surgery; Fang et al. [18] found that the proportion of overestimation of lenticule thickness in predicted value is 11.9% for ultrasound and about 15% for Pentacam. However, previous studies were only limited to studying the overestimation rate of nomogram and obtained a more accurate estimate value by reducing a certain proportion of nomogram; few studies had constructed a model to predict LT from the major factors affecting LT. Therefore, our study focuses on exploring the major factors that affect LT and predict LT by multiple linear regression and various machine learning models to finally obtain an optimal prediction model.

The precise prediction of LT was according to biometric parameters, including age, sex, CCT, LD, K-mean, E, S, Cyl, and AL. During the study, we gradually excluded the effects of AL, age, sex, and K-mean on LT by Pearson correlation analysis and multiple linear regression models and accidentally found that E and CCT had significant effects on LT, which had not been mentioned in previous studies. With the development of AI, machine learning has been widely used in the medical field. We applied machine learning to predict LT and found that the accuracy of the Random Forest model in predicting LT was higher than that of the multiple linear regression model, and further confirmed the significance of CCT and E in the prediction of the model. Despite the small sample in this study, we found high accuracy of LT prediction using the multiple linear regression model and some machine learning models.

Then, we selected 50 subjects as the validation data to verify the prediction accuracy of the model. It was found that the nomogram tended to overestimate the value of LT, with an average overestimate of 19.59%, while the Random Forest model had a much higher prediction accuracy, with an average underestimate of −0.15%. It was good news for some patients with high refractive errors but thin CCT because they could get correction as much as possible, which is of great significance for the improvement of visual quality and satisfaction of patients after surgery. However, the Random Forest model also has its limitations; it tends to overestimate when LT is thin and tends to underestimate when LT is thick, which may cause postoperative residual CCT shortage caused by long-term complications, such as corneal expansion [32]. Therefore, we need to further expand the amount of data to improve the accuracy of our model prediction in the future, and longer follow-up would be needed to better understand the changes rule of residual CCT and its impact on the corneal health after SMILE surgery in the meantime.

## 5. Conclusions

The results of this study validate the reliability of machine learning models in accurately predicting LT in SMILE surgery and screen out the best-performing Random Forest model. In addition, we found two factors significantly affecting the prediction of LT; they are preoperative CCT and anterior corneal surface eccentricity (E), respectively. Furthermore, the nomogram from VisuMax femtosecond laser system significantly overestimates the achieved LT, while based on the Random Forest model, we are able to obtain closer prediction results to the achieved LT.

## Figures and Tables

**Table 1:** Basic information and ocular parameters of the subjects included in this study.

Subjects	Values	
No. of cases	352	
Sex, male No. (%)	189 (53.69)	
Sex, female No. (%)	163 (46.31)	
Variables	Range	Mean ± SD ^9^
Age (years)	18–48	22.87 ± 5.92
K-mean ^1^ (D ^8^)	39.1–45.8	42.91 ± 1.30
CCT ^2^ (μm)	491–656	554.57 ± 28.75
E ^3^	0–0.83	0.56 ± 0.097
LD ^4^ (mm)	6.0–6.8	6.51 ± 0.17
S ^5^ (D ^8^)	−10–−1.5	−5.75 ± 2.15
Cyl ^6^ (D ^8^)	−3.75–0	−0.96 ± 0.74
AL ^7^ (mm)	23.33–29.90	26.17 ± 1.07
No. of cases	352	

^1^ K-mean: mean K reading; ^2^ CCT: central corneal thickness; ^3^ E: eccentricity of the anterior corneal surface; ^4^ LD: lenticule diameter; ^5^ S: Sphere; ^6^ Cyl: Cylinder; ^7^ AL: axial length; ^8^ D: diopters; ^9^ SD: standard deviation.

**Table 2:** Performance of the multiple linear regression model and machine learning models.

	Models	R^2^	R	MSE ^5^	RMSE ^3^	MAE ^4^
Linear Model	Multiple Linear Regression	0.87	0.93	87.44	9.35	6.15
Machine Learning Methods	Logistics Regression	0.66	0.81	229.07	15.14	11.15
	KNN ^2^	0.74	0.86	184.37	13.58	10.23
	SVM ^1^ (linear)	0.80	0.89	139.11	11.79	7.68
	Decision Tree Regression	0.88	0.94	80.06	8.95	2.48 *
	Ridge Regression	0.87	0.93	90.72	9.52	6.46
	Bayesian Linear Regression	0.87	0.93	90.15	9.49	6.42
	Lasso Regression	0.87	0.93	89.91	9.48	6.41
	Random Forest	0.95 *	0.98 *	32.66 *	5.71 *	3.78

^1^ SVM: Support Vector Machine; ^2^ KNN: K-Nearest Neighbor; ^3^ RMSE: root mean square error; ^4^ MAE: mean absolute error; ^5^ MSE: mean squared error. Best values of indices are marked by an asterisk (*). Keep two significant digits after the decimal point.

**Table 3:** Variables included in the prediction model.

Variables	Coef ^6^	SE ^7^	t	*p*	95%CI ^8^
S ^1^	−11.26	0.30	−37.27	0.000 **	−11.86, −10.67
Cyl ^2^	−12.17	0.78	−15.68	0.000 **	−13.70, −10.64
LD ^3^	29.40	4.09	7.18	0.000 **	21.34, 37.45
CCT ^4^	0.046	0.021	2.18	0.030 *	0.004, 0.087
E ^5^	−12.19	5.836	−2.09	0.038 *	−23.68, −0.71

^1^ S: Sphere; ^2^ Cyl: Cylinder; ^3^ LD: lenticule diameter; ^4^ CCT: central corneal thickness; ^5^ E: eccentricity of the anterior corneal surface; ^6^ Coef: coefficient; ^7^ SE: standard error; ^8^ CI: confidence Interval; * indicates that the *p*-value less than 0.05; ** indicates that the *p*-value less than 0.01.

**Table 4:** Variables that included in the prediction model.

Model	Group 1	Group 2	Group 3	Group 4	Group 5	Average
Nomogram	22.11%	19.21%	22.98%	16.00%	17.64%	19.59%
RF model	7.51%	−0.26%	0.78%	−5.19%	−2.99%	−0.15%

RF: Random Forest.

## Data Availability

The datasets used and/or analyzed during the current study are available from the corresponding author upon reasonable request.
